# Dataset for carbon footprints of transforaminal lumbar interbody fusions performed under spinal or general anesthesia

**DOI:** 10.1016/j.dib.2022.108218

**Published:** 2022-04-27

**Authors:** Andy Y. Wang, Tameem Ahsan, Jacob J. Kosarchuk, Penny Liu, Ron I. Riesenburger, James Kryzanski

**Affiliations:** a Department of Neurosurgery, Tufts Medical Center, Boston, MA, USA; b Department of Anesthesiology, Tufts Medical Center, Boston, MA, USA

**Keywords:** Environment, Environmental impact, Spine, Spinal anesthesia, Orthopedic surgery, Neurosurgery

## Abstract

The datasets presented here quantify and compare the relative carbon footprints emitted by general versus spinal anesthesia in patients undergoing single-level transforaminal lumbar interbody fusions (TLIFs). Data were retrospectively collected from electronic medical records of 100 consecutive patients who underwent a single-level TLIF from a single neurosurgeon at a U.S. academic center. 50 patients were under general anesthesia, and another 50 patients were under spinal anesthesia. Clinic and operative notes were used to extract demographic and surgical information, whereas anesthesia records were used to extract anesthetic information. Using the anesthetic information, carbon dioxide equivalents (CO_2_e) were calculated for each type of anesthetic and summed together to compute the total carbon footprint for each patient. Our article entitled “Assessing the environmental carbon footprint of spinal versus general anesthesia in single-level transforaminal lumbar interbody fusions” is based on this data [1]. Raw datasets of the primary data collection as well as cleaned and analyzed datasets are presented. These datasets highlight the first known environmental impact calculation from medical records of a spine procedure, serving as a model for other interested investigators to explore and emulate. This data brief may help to pave the way towards future environmental research and practice changes within neurosurgical and orthopedic literature, an issue critical to the sustainability of our modern society.

## Specifications Table

**Table:** 

Subject	Surgery
Specific subject area	Spine surgery (neurosurgery and orthopedic surgery) and environmental impacts
Type of data	Excel spreadsheets R code
How the data were acquired	Data was acquired through retrospective review of medical, surgical, and anesthesia records at Tufts Medical Center through the Soarian electronic health record system.
Data formats	Raw Analyzed and cleaned
Description of data collection	The research team collected data from 100 consecutive patients who underwent a single-level TLIF from a single surgeon at an academic center through a retrospective chart review of the electronic medical records. Baseline characteristics were gathered from clinical and operative notes. Every anesthetic agent used during each surgery was recorded from the intraoperative anesthesia records.
Data source location	• *Institution: Tufts Medical Center* • *City/Town/Region: Boston, Massachusetts* • *Country: United States of America* • *Latitude is 42.3601° and Longitude is −71.0589°*
Data accessibility	Repository name: Mendeley Data Data identification number: https://doi.org/10.17632/npyyj7s854.1 Direct URL to data: https://data.mendeley.com/datasets/npyyj7s854/1
Related research article	A.Y. Wang, T. Ahsan, J.J. Kosarchuk, P. Liu, R.I. Riesenburger, K. Kryzanski, Assessing the environmental carbon footprint of spinal versus general anesthesia in single-level transforaminal lumbar interbody fusions, World Neurosurgery. In Press.

## Value of the Data

• These datasets provide carbon footprint calculations for the environmental impact of spine surgery anesthetic modalities and serve as a model workflow for future research.
• These datasets allow for the critical appraisal of comparisons of environmental impact between the use of general versus spinal anesthesia.
• Neurosurgeons and orthopedic surgeons can benefit from these data and better understand the results of our study, including the impact of a change in surgical practice, or to perform further research understanding the environmental impacts of spine surgery.
• These data can be used and reused for further explorations, such as starting a database of environmental impacts of spine surgeries or exploring the nuances of the carbon footprint of different types of anesthetics.

## 1 Data Description

“demo_nongas.xlsx” is the Excel workbook that contains basic demographic information, amounts of each type of non-inhaled anesthetic used, conversion of anesthetic type to carbon dioxide equivalents (CDE), and the sum of CDE equivalents for each patient. [PATIENT_ID] is the unique identifier for each patient. [SPINAL_GETA] refers to whether spinal or general anesthesia was used. [GENDER] refers to the gender of the patient, [AGE] refers to the age at surgery, and [BMI] refers to the body mass index of the patient, and [ASA] refers to the American Society of Anesthesiology score. [SURGERY_INDICATION] refers to the reason for surgery, [SURGERY_TYPE] details specifically what was performed, [LEVELS] refers to the operative levels. Columns J to R refer to the different time components of the operative timeline. Columns S to BU refer to the different quantities of anesthetics used during the surgery along with a unit of measurement. Columns BX to CQ refer to the calculations of carbon dioxide equivalents of each type of anesthetic used. [OTHER_CDE] refers to the sum carbon dioxide equivalent total of all non-gaseous anesthetics used for each patient.

In the folder “Gases,” are Excel spreadsheets numbered “1.xlsx” to “50.xlsx,” corresponding to the individual anesthetic records of each patient. Though each patient has varying numbers of rows for each patient, the top group of rows correspond to the use of each type of anesthetic at each time interval column. The bottom rows correspond to volume calculations derived for each gaseous anesthetic at timepoint, summed together to a final volume at the rightmost cell of the row.

“gas.xlsx” is the Excel workbook that contains information for inhaled anesthetics, including the quantity of gaseous anesthetics and their associated carbon footprints as calculated from spreadsheets in the “Gases” folder. [PATIENT_ID] and [SPINAL_GETA] is the same as the other dataset. [DESFLURANE_L], [ISOFLURANE_L], [SEVOFLURANE_L] and [N2O_L] refers to the liters of each type of inhaled anesthetic used throughout the surgery. [DES_CDE], [ISO_CDE], [SEVO_CDE], and [N2O_CDE] refer to the carbon dioxide equivalents of each type of anesthetic for each patient. [OTHER_CDE] is the same as that of the other dataset, carried over. [TOTAL_CDE] is the sum of all gaseous and non-gaseous anesthetics for each patient, which is the total carbon footprint for each patient.

“environment.R” is the R code that can be used to analyze the data.

## 2 Experimental Design, Materials and Methods

The data presented here aims to quantify and compare the relative carbon footprints emitted by general versus spinal anesthesia in patients undergoing single-level transforaminal lumbar interbody fusions (TLIFs). The retrospective study involved 100 consecutive patients who each underwent a single-level TLIF from a single surgeon at an academic center. 50 patients were under general anesthesia, and 50 were under spinal anesthesia. Any patient with additional surgical steps beyond a simple single-level TLIF was excluded. Retrospective chart review of electronic health records was performed to extract baseline and surgical characteristics, as well as anesthetic information. The anesthetic flowsheet of each type of anesthetic used at each timepoint of the surgery was recorded from the intraoperative anesthesia records.

To calculate the amount of each type of non-gaseous anesthetics used for each patient, the amounts of each type of anesthetic across all time periods were summed into a single value. For gaseous anesthetics, the volume of anesthetic was calculated using this equation: Gas (L) = Time * Free gas flow (L/min) * End-tidal gas concentration (%) [2]. This was performed at every 15-minute time interval in the anesthesia flowsheet, and then summed to provide a total volume for each type of anesthetic.

To calculate the carbon footprint of each type of anesthetic in carbon dioxide equivalents (CO_2_e), a conversion unit called global warming potential (GWP) is used. GWP describes how much energy the emissions of one mass of a type of gas will absorb over a given time (usually 100 years), relative to the energy the same mass of carbon dioxide will absorb (GWP = 1). Therefore: CO_2_e (g) = Anesthetic agent (g) x Anesthetic GWP_100_. The values for molar mass, density, and GWP_100_ of each type of gaseous and nongaseous anesthetic has been previously determined by prior research [3], [4], [5]. With this equation, the calculations for nongaseous anesthetics are simple as the mass of each type of anesthetic is readily available from patient charts. In order to calculate the mass of gaseous anesthetics, the volume must be converted with the following equation: Anesthetic agent (g) = Gas (L) * Molar mass (g/mol) ÷ (2412 * Density) [6].

## Ethics Statements

Research was carried out in accordance with the Declaration of Helsinki and approved by the Tufts Medical Center's Institutional Review Board (MOD-01-STUDY00002002). Requirement for consent was waived for this retrospective study.

## CRediT Author Statement

**Andy Y. Wang**: Data curation, Validation, Visualization, Formal analysis, Investigation, Roles/Writing - original draft, Writing - review & editing. **Tameem Ahsan:** Data curation, Validation, Investigation, Writing - review & editing. **Jacob J. Kosarchuk:** Investigation, Data curation, Writing - review & editing. **Penny Liu:** Resources, Writing - review & editing. **Ron I. Riesenburger:** Conceptualization, Resources, Supervision, Writing - review & editing. **James Kryzanski:** Conceptualization, Resources, Supervision, Writing - review & editing.

## Declaration of Competing Interest

The authors declare that they have no known competing financial interests or personal relationships that could have appeared to influence the work reported in this paper.

## Data Availability

Dataset for carbon footprints of transforaminal lumbar interbody fusions performed under spinal or general anesthesia (Original data) (DIB).

## Appendix Supplementary materials

Image, application 1

## References

[1] A.Y. Wang, T. Ahsan, J.J. Kosarchuk, P. Liu, R.I. Riesenburger, J. Kryzanski, Assessing the environmental carbon footprint of spinal versus general anesthesia in single-level transforaminal lumbar interbody fusions, World Neurosurg.. In Press. 10.1016/j.wneu.2022.03.095 35342029

[2] Ryan S.M., Nielsen C.J. (2010). Global warming potential of inhaled anesthetics: application to clinical use. Anesth. Analg..

[3] Parvatker A.G., Tunceroglu H., Sherman J.D., Coish P., Anastas P., Zimmerman J.B., Eckelman M.J. (2019). Cradle-to-Gate Greenhouse Gas Emissions for Twenty Anesthetic Active Pharmaceutical Ingredients Based on Process Scale-Up and Process Design Calculations. ACS Sustain. Chem. Eng..

[4] Sherman J., Le C., Lamers V., Eckelman M. (2012). Life cycle greenhouse gas emissions of anesthetic drugs. Anesth. Analg..

[5] Sulbaek Andersen M.P., Nielsen O.J., Wallington T.J., Karpichev B., Sander S.P. (2012). Medical intelligence article: assessing the impact on global climate from general anesthetic gases. Anesth. Analg..

[6] Dion P. (1992). The cost of anaesthetic vapours. Can. J. Anaesth..

